# Which Traits of Humic Substances Are Investigated to Improve Their Agronomical Value?

**DOI:** 10.3390/molecules26030760

**Published:** 2021-02-02

**Authors:** Heejung Jung, Sumin Kwon, Jae-Hwan Kim, Jong-Rok Jeon

**Affiliations:** 1 Department of Agricultural Chemistry and Food Science & Technology, Division of Applied Life Science (BK21Plus), and IALS, Gyeongsang National University, Jinju 52828, Korea; gksmf8797@naver.com (H.J.); mych1800@naver.com (S.K.); 2 Advanced Geo-materials R&D Department, Korea Institute of Geoscience and Mineral Resources, Pohang 37559, Korea

**Keywords:** humic substance bioactivity, sustainable agriculture, plant stimulants, structure–property–function relationship

## Abstract

Humic substances (HSs) are chromogenic organic assemblies that are widespread in the environment, including soils, oceans, rivers, and coal-related resources. HSs are known to directly and indirectly stimulate plants based on their versatile organic structures. Their beneficial activities have led to the rapid market growth of agronomical HSs. However, there are still several technical issues and concerns to be addressed to advance sustainable agronomical practices for HSs and allow growers to use HSs reliably. First, it is necessary to elucidate the evident structure (component)–function relationship of HSs. Specifically, the core structural features of HSs corresponding to crop species, treatment method (i.e., soil, foliar, or immersion applications), and soil type-dependent plant stimulatory actions as well as specific plant responses (e.g., root genesis and stress resistance) should be detailed to identify practical crop treatment methodologies. These trials must then be accompanied by means to upgrade crop marketability to help the growers. Second, structural differences of HSs depending on extraction sources should be compared to develop quality control and assurance measures for agronomical uses of HSs. In particular, coal-related HSs obtainable in bulk amounts for large farmland applications should be structurally and functionally distinguishable from other natural HSs. The diversity of organic structures and components in coal-based HSs must thus be examined thoroughly to provide practical information to growers. Overall, there is a consensus amongst researchers that HSs have the potential to enhance soil quality and crop productivity, but appropriate research directions should be explored for growers’ needs and farmland applications.

## 1. Humic Substances for Agronomical Uses

Humic substances (HSs) are chromogenic and structurally irregular organic assemblies that are widespread in soils, rivers, oceans, and coal-related natural resources (i.e., peat, leonardite, and lignite) [1,2]. HSs are believed to be derived from dead living organisms of mainly plant origin [1,3]. Once the organic substances are released into the environment, the recalcitrant components can be maintained via their structural irregularities and mineral complexations [4]. Since several organic moieties found in HSs are involved in redox mediating, electron donating/accepting, adhesion, and acidity [4], HSs directly induce several biotic and abiotic reactions contributing to microbial respiration, soil fertility, xenobiotic transformation, and metal bioavailability [4,5]. In particular, use of HSs to enhance crop productivity and soil quality is of great interest owing to the recent need for eco-friendly organic fertilizers over traditional NPK (nitrogen, phosphorus, and potassium)-based chemical fertilizers [6]. Although HSs hardly contain plant-available NPK nutrients [1,4], their roles as soil amendments and plant stimulants are supported by enhanced plant growth [4,6]. This trend is also associated with the continuous loss of soil organic carbon in farmlands, thereby requiring artificial addition of organic carbons to obtain organically rich soils. [7]. HSs’ ability to increase soil organic carbon sequestration indicates that artificially supplied HSs contribute to hampering the loss of organics in soils [8]. 

HS treatments have been shown to have beneficial effects on crop productivity as they contain a variety of oxygen-based functional groups, which render soil environments relatively acidic [4,5]. Macronutrients such as phosphorous become more available to plants because of the higher solubility of phosphorous-containing particles to acidic conditions [9,10]. The polymeric features of HSs and the inherent adhesive actions attributable to the oxygen-based functional groups allow soil particle aggregation, which is relevant to crop productivity [11,12]. Indeed, soil-type- and HS extraction source-dependent aggregation patterns have been observed [11]. Interestingly, HSs are capable of directly stimulating plants by modulating their gene and functional protein expressions [4,5]. Scientific clues proving penetration of HS components in plant roots have been demonstrated with microautoradiography [13]. Although detailed mechanisms of how HSs overcome the plant rhizoplane remain to be elucidated, they seem to be dependent on the molecular size distributions of HS components [5]; once penetrated, the transportation from roots to shoot was proven feasible via transpiration [13]. Some functional protein activities, including high-affinity K^+^ transporter 1, phospholipase A2, and H^+^-ATPase, are known to be modulated by HSs [4,14]. More research is needed to identify how HS components affect such enzymes’ activities; however, two plausible mechanisms may be considered here. First, oxygen-based functional groups in the HS induce pH changes in the cell surroundings, thereby causing changes in membrane protein activities. Specifically, alkalization of root cell membranes by the action of H^+^/NO_3_^−^ symporter could be counteracted with HS acidity [4,5]. Second, the non-specific adhesive properties of HSs are likely to interact with functional proteins, which may either be activated or deactivated. This hypothesis was also substantiated by the experimental evidence showing HSs’ ability to physically encapsulate proteins via electrostatic interactions in an in vitro manner [4]. 

Beneficial actions of HSs in the agricultural domain have mainly been evaluated by academic researchers and linked to rapid growth of the commercial market for HSs. The market size for humic acids is estimated to show over a 14% compound annual growth rate (CAGR) from USD 510 million in 2018, while that for fulvic acids was approximately USD 228 million in 2019, with a 3.5% CAGR [15,16]. The major market areas for HSs include agriculture and horticulture. Nevertheless, there is still a certain skepticism among growers regarding the effectiveness of HSs on crop productivity and marketability. To this end, several academic studies have reported meager enhancements or even adverse effects of HS. Hartz and Bottoms showed that fruit yields and biomass weights were not enhanced in response to HS addition to soils [17]. Suddarth et al. also suggested that combined treatment of a HS with irrigation might increase the salt content of soils [18]. Therefore, imminent discussions are necessary to direct HS research and development toward more practical and reliable agronomic recommendations. We thus suggest two points of view regarding crop growers and HS types. It was here discussed which aspects of HS modes of action and application are further investigated to satisfy crop growers and which types of HS among those from different extraction sources should be preferred for their agronomical application research.

## 2. Plant Multi-Stimulatory Actions of HS: Need for Satisfying Crop Growers

It has been demonstrated that HSs regulate several plant physiological processes including seed germination, root genesis, and abiotic stress resistance [4,5,14,19]. Herein, we summarize the structural features of HSs that are required for their specific plant stimulatory actions. 

HSs play critical roles in seed germination acceleration. Seed germination of several plant species such as maize [20], *Arabidopsis thaliana* [10,21], and rice [22] was proven to be facilitated in the presence of an HS. Phenolic moieties of the HS structures are regarded as the main factor contributing to seed germination acceleration [20]. Interestingly, low-molecular-weight phenolics, such as *p*-coumaric acid and *p*-hydroxybenzoic acid, which are widespread in soils, exhibit strong inhibitory actions on seed germination by hampering the glycolysis and oxidative pentose phosphate pathways [23], while polymerized phenolics that are structurally similar to HSs stimulate seed germination [21]. These contrasting properties are dependent on the extent of polymerization and suggest that HS molecular weights critically affect the extent of phenol-based enzyme inhibition. This opinion is further evidenced by the fact that HS treatments over specific concentrations inhibit seed germination [21]. 

Root development is likely to be enhanced in HS-treated plants. Particularly, lateral root proliferation appears to be strongly enhanced [21,24,25]. There are three mechanisms that explain root stimulation. Firstly, hormone-like organic structures such as auxin activate specific hormone pathways in plants; the auxin structure was identified with organic moieties of HS isolated from earthworm compost [24], but experiments using HS analogs synthesized by polymerization of known small phenols deficient in detailed auxin structures still suggest that the overall distribution of specific organic functional groups, including phenols and carboxylic acids, of HSs are more important [21]. More specifically, the structural characteristics between root genesis and growth are distinguishable. As reported in Garcia et al., labile and functional organic groups are more involved in root genesis, while recalcitrant and less functionalized ones induce root elongation [26]. Structural features stimulating only root elongation have recently been reported. The aliphatic OH of lignin was proven to be crucial for inducing maize seedling development while leaving the germination rate unaffected [27]. Secondly, nitric oxide is produced in the presence of HS, followed by lateral root development. The fact that nitric oxide scavengers, rather than auxin inhibitors, effectively hamper HS-driven root development indicates that auxin-independent pathways are activated by HSs [25]. Thirdly, energy metabolism is enhanced with increased expression of the related proteins [28]. Facilitation of energy acquisition may thus be linked to root cell proliferation. The latter two modes of action suggest that the key biological factors (i.e., nitric oxide and energy-metabolism-related proteins) are required to change root genesis, though the identification of the key structural moieties of HSs to activate such factors has not yet been investigated. 

HS treatments are believed to allow plants to resist abiotic stresses such as excessive heat and salts in the growth media more efficiently. Economically important vegetables such as pepper, tomato, and watermelon were tested regarding HS effectiveness against heat stress [29,30]. The results showing enhancement of crop productivity support that HSs stimulate heat defense mechanisms at the molecular level. Cha et al. recently showed that a class of heat shock protein transcripts are upregulated with HS treatment and this activation coincides with enhancement of plant survival rates, suggesting that the transcripts are translated into functional proteins followed by plant phenotypic changes [31]. However, HS organic structures for heat stress-related plant priming have not yet been evaluated. It is also questionable whether HS-driven molecular activation pathways for heat resistance are distinct from those of root stimulations.

HS-driven alleviation of salt stress also has similar research patterns to heat stress, except for the use of artificially synthesized polymers. Plants including cress, maize, and *A. thaliana* have been shown to be activated by HSs, thus coping with salt stress [21,32]. In general, the stress-induced hampering of seed germination, chlorophyll maintenance, and plant biomass increase are found to be mitigated. It is observed that similar organic moieties such as phenols and carboxylic acids found in seed germination are also applicable to improve plant resistance to salt stress [21]. This indicates that the activation pathways of seed germination and salt resistance are shared. Specific molecular targets are also suggested; for instance, high-affinity K^+^ transporter 1, involved in sodium influx, is thought to be stabilized in the presence of HSs [14]. 

Another aspect to consider in HS-induced plant stimulations is the HS modes of application, because addition to soil, root immersions, and use of foliar sprays are the options available to treat plants with HSs. Hita et al. reported the possibility to elicit different cucumber responses regarding the treatment methods (i.e., root immersions in hydroponic solutions and foliar applications) [33]. Both treatments induced shoot and root growth, but the root plasma membrane H^+^-ATPase activity was enhanced only with root applications, while jasmonic acid production in the shoot was enhanced only with foliar applications. Immersion of plant tissues also shows different HS stimulations. Lilium cultivation treated with HSs in immersion solutions resulted in increased plant nutrient accumulation and sugar synthesis [34]. On the contrary, anthocyanin accumulation was likely to be induced with the spray method [34]. These results suggest that crop productivity could be significantly affected according to the modes of HS application.

It has been proven that mineral and organic matter contents differ across soil types [35,36]. Owing to this versatility, HS soil applications may induce different types of plant stimulations. Naturally occurring soil minerals non-covalently interact with HS fractions [37], which would result in the modulation of HS activities for plant stimulations. For instance, apatites present in some soils could be surface-engineered by HSs, thus accelerating phosphate release, which improves plant nutrition [10]. The current authors indeed found that a simple dipping of apatite particles in HS solutions allows for their surface modification, which is linked to accelerated phosphate release [10].

Soil organic matter qualities and quantities are versatile across soil types and land uses [36]. There is a possibility that soils rich in organic matter show less effectiveness with exogenous HSs because the stimulatory action could be already assured by the native soil HSs. This was evidenced by the findings that the microbial activity of low organic matter soils is more enhanced with HSs than that of high organic matter soils [17]. In addition, different soils induce different HS behaviors because the self-assembly of HS components and their solubility are dependent on the pH, ions, and oxidative potentials of the surroundings. Specifically, Celik et al. proved the HS function in the enhancement of weight increase and uptake of several nutrients for plants grown in calcareous soils [38]. Hartz et al. also showed that plant dry weight increase with HSs depends on specific soil textures [17]. Accordingly, the prevailing soil conditions determine the effective actions of HS on plants.

As described before, it is possible to anticipate that HS stimuli on plants occur in multifunctional ways, but the current results, primarily obtained in lab conditions, are far from being translated to crop field recommendations, so they hardly fulfill the needs of farmers. Herein, we suggest three methods to facilitate HS applications in farmlands by growers.

Artificial structure engineering in a bottom-up manner may provide the synthesis of humic-like substances in which specific organic functional moieties are present or absent [21]. The extent of plant stimulation by artificial HS analogs with different structural characteristics will shed light on the key HS structures capable of inducing specific plant physiological changes. Such trials may accelerate the discovery of alternative and renewable natural resources to produce humic-like substances containing crucial plant-stimulating organic moieties. Moreover, natural or semi-synthesized HS analogs showing superior plant stimulatory actions than natural HSs could be suggested. In fact, plant lignin and hydrochar were found to be valuable materials for obtaining humic-like substances capable of stimulating plants [20,39]. Composting using vegetable, plant residues and animal manures was also proven to lead to the production of HSs [40]. When both times for complete reaction/extraction and costs of raw materials and reaction/extraction agents become minimized, these approaches decrease HS consumer prices, thus allowing growers to utilize HSs at technically higher rates. Most HSs for agronomical purposes are currently from coal-related materials [2], which are biased to specific and a few territory regions. This situation may increase local or domestic HS prices for their on-site uses. Indeed, the current authors identified, through personal discussion with domestic HS vendors, that the most limiting factor to the large-scale use of HSs in the Republic of Korea is their high price to farmers. 

Further studies should focus on elucidating whether crop characteristics relevant to the market values change with HS treatments because most studies have dealt with several physiological changes of plants that are not clearly expected to enhance crop marketability and farmer profitability. For instance, several reports indicate that plant seed germination is accelerated in the presence of HSs [20,21,22], but little is known about whether this enhancement results in better food yield and quality. Plant root stimulations, including omics-based action mechanisms of HS, are frequently handled by researchers [28], but it still remains to be elucidated whether such stimulations are generally connected to enhanced crop productivity that allows growers to derive more profits. It will engage more attention from growers to the use of HSs if the treatments are tightly associated with crop marketability. It is also necessary to confirm whether HS experimental conditions to enhance crop marketability at a lab scale are applicable and reproducible in a field test, where farmers are engaged. In fact, optimal HS treatment concentrations between laboratory and greenhouse plant stimulation assays were found to be different [41].

**Figure 1:**
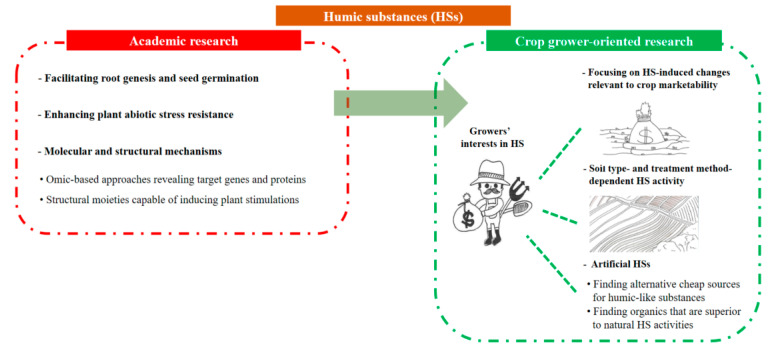
A need for satisfying crop growers to boost use of humic substances (HSs) in farmlands. Previous academic results and further studies of HSs should be reinterpreted and performed, respectively, in terms of helping crop growers.

Although soil environments are likely to dramatically affect HS effectiveness, comparative studies in terms of soil types and textures are lacking. Soil factors such as pH, pre-existing organic matter content, porosity, electrical conductivity, and availability of nutrients could, thus, be assessed together. This allows growers to compare their own soil types with a data pool of scientifically collected and validated HS effectiveness information. Moreover, such soil characteristics of fields could be a critical factor for the reproduction of HS lab-scale performances in farmland fields. Figuring out the relationship between soil characteristics and plant stimulatory actions of artificially added HSs will accelerate HS uses by farmers while achieving enhancement of crop marketability at the field scale. Likewise, comparative research using different crops and modes of HS application should be more intensively conducted to allow growers to select proper treatment methods for their large farmlands. Research and development directions to attend to farmers’ needs are summarized in Figure 1.

## 3. HSs for Agronomical Use: Elucidating Coal-Derived HS Properties and Functions

HSs are irregular organic structures whose molecular formulas are hard to define. Rather, specific HS extraction methods from the environment such as soils, rivers, and oceans determine detailed HS compositions, and it is thus likely that organic characteristics and their deposition history in the environment where HSs are extracted are the most important factor affecting the detailed humic components extracted [1,4]. Hence, HSs’ structural differences are necessarily observed with HS extraction sources and use of different extraction methods. For example, detailed elemental composition and aliphatic/aromatic ratios of HS are found to significantly vary depending on the HS extraction protocols and sources. In particular, HSs from soils and rivers exhibit different structural features than coal-related commercial HSs, considering that a strong 13C NMR shift near 170 ppm and a higher O/C ratio of element analysis are observed in HSs from soils and rivers compared with coal HSs [42]. These results suggest that crop stimulations and soil quality improvements by coal-related and soil-/river-extracted HSs should be evaluated in terms of different structural views. In particular, a systemic structural comparison between coal- and other environment-derived HSs seems to be urgently investigated.

Many research studies have investigated soil-derived, vermicomposted, or plant-derived HSs to evaluate HS structure–function relationships for plant stimulations [20,24,25,26,27,28,39]. However, in reality, it is difficult to use soil-extracted HSs for large-scale applications because the extractable HS quantity is relatively small and limited. Vermicomposted (or composted) and plant-derived substances are also based on chemical (or biological) transformation and extraction of raw materials, which still appear to be time-consuming and economically non-viable [24,27,39,40]. Indeed, most commercial products currently available for agronomical purposes are obtained from non-renewable coal matrices, such as peat, leonardite, and lignite [2,4], which raises questions as to whether key structural aspects associated with plant stimulations are also identifiable in coal-related HSs. Moreover, it is plausible that geological differences may result in detailed component variations of coal materials, thereby affecting action modes of HS components. Comparative studies to visualize composition variations of diverse coal HSs should thus be conducted to encourage growers to utilize coal HSs for crop cultivation reliably. Contrary results of crop productivity by coal-derived HSs (i.e., enhancement or meager effects) [17,18] can be re-interpreted in terms of the structural views of such materials.

Coal-related HSs are known to contain more than organic substances. Particularly, the inherent presence of Fe species seem to be significant [39] and thus considerable because they are able to perform dual functions. First, Fe can be used as a plant macronutrient. Second, Fe species are capable of interacting with HS components, thus changing the HS component-involved supramolecular structures. This conformational change is likely to tune the plant Fe nutrition and HS ability to penetrate plant roots [43]. Owing to these reasons, artificial Fe was even added to coal HSs to boost plant stimulation and Fe nutrition in alkaline soils [44]. Therefore, inherent metallic substances beneficial for plant nutrition should be assessed to differentiate coal-derived HSs from other types of HS. 

Another aspect that should be considered is the inherent presence of microbes in coal-related HSs. Various microbes are present in peat, leonardite, and lignite. Coal-related HSs could, thus, contain microbes [2], which, in turn, spontaneously extend to co-treatments of HS organic components and the microbes to crops and farmlands. In fact, plant-stimulating endophytic microbial communities can be induced with coal-related HSs in hydroponic systems, suggesting that different microbial communities of plants result from HS treatments [45]. More direct evidence was obtained by a direct isolation of plant-stimulating microbes from some commercial coal-related HS powders [2]. Therefore, it is worth quantitatively estimating the microbial activities in HSs that are relevant to crop stimulations. These inorganic and biological factors could be exploited to promote more reliability for the use of coal-related HSs by crop growers.

**Figure 2:**
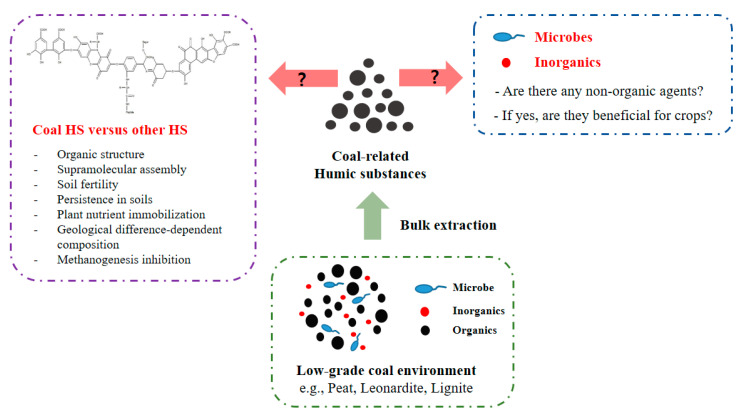
A scheme for coal HS-oriented research. Coal HS is one of the solutions to apply in large farmlands and their unique characteristics (e.g., structural differences and potential presence of non-organic agents) that are distinctive from other HSs should be thus unraveled to give technical reliability to crop growers. The assumed organic structure of HS is from Vekariya et al. (2016) [49].

It is believed that soil fertility depends heavily on the organic content in the soil, which is vital for the maintenance of plant inorganic nutrients and soil fertility [7]. Soil organic matter decomposition has been reported owing to agriculture involving tillage and irrigation [46,47]. It is generally accepted that the solution to this problem is the artificial addition of plant-derived organics into soil-based environments [7,8]. To this end, coal-related HSs capable of treating large areas of soil farms with significant amounts of addition could be key to replenishing organic matters in arid soils. HSs’ supramolecular assembly recalcitrance to microbial catabolism allows them to persist in soils for long periods of time [4,8]. Moreover, versatile oxygen-based functional groups in HSs act as chelating sites to prevent leaching of several plant metallic nutrients [1,4]. Managing greenhouse gas emission (GHE) from agriculture domain is recently of interest. HS ability to accept electrons involved in methanogenesis results in methane gas emission mitigation in peats [48]. Agronomical value of HSs will be higher when HS treatments in farmlands such as paddy fields result in GHE mitigation which is further subjected to the GHE trading. Accordingly, coal HS performances should be assessed in terms of the soil organic functionalities compared with those of HSs derived from other sources. Positive coal-derived HS effects as soil organics in long-term use could, thus, attract attention from growers to facilitate continuous use of HSs in farmlands. Research and development directions for coal HS are depicted in Figure 2.

## 4. Concluding Remarks

Many researchers have demonstrated that HSs are effective for enhancing both soil fertility and plant nutrition and growth. Despite the use of advanced analytical tools to unveil HS modes of action on plants, there are still concerns from growers on whether HSs are truly effective for increasing crop yield. New research and development directions are thus necessary to enable HSs as eco-friendly and irreplaceable materials for sustainable agricultural practices while transferring technologies and improving profitability to crop growers. Therefore, crop species, treatment methods (i.e., roots, foliar, or immersion applications), and soil type-dependent HS plant-stimulatory actions should be detailed, and the related core organic structures should be unraveled. Moreover, HS-based solutions to induce changes in crop physiological processes connected to marketability and food quality need to be provided to crop growers. Additionally, studying coal-derived HSs that can be used on a large scale in crop fields should be intensified. Other components such as microbes and metals identifiable in coal-related HSs could be considered in terms of their benefits to improve soil quality and crop productivity. These research directions will pave the way for boosting HS applications in sustainable agriculture while offering credibility to the growers.

## Data Availability

The data presented in this study are available on request from the corresponding author.
